# Targeting activated PI3K/mTOR signaling overcomes acquired resistance to CDK4/6-based therapies in preclinical models of hormone receptor-positive breast cancer

**DOI:** 10.1186/s13058-020-01320-8

**Published:** 2020-08-14

**Authors:** Neil A. O’Brien, Martina S. J. McDermott, Dylan Conklin, Tong Luo, Raul Ayala, Suruchi Salgar, Kevin Chau, Emmanuelle DiTomaso, Naveen Babbar, Faye Su, Alex Gaither, Sara A. Hurvitz, Ronald Linnartz, Kristine Rose, Samit Hirawat, Dennis J. Slamon

**Affiliations:** 1 Department of Medicine, Division of Hematology/Oncology, Geffen School of Medicine at UCLA, Los Angeles, CA USA; 2 Novartis Pharmaceuticals, Cambridge, MA USA; 3 Currently Bayer Pharmaceuticals, Boston, MA USA; 4 Currently LG Life Sciences, Cambridge, MA USA; 5 Currently Bristol Myers Squibb, Lawrenceville, NJ USA; 6 UCLA Translational Oncology, 2825 Santa Monica Blvd, Suite 200, Santa Monica, CA 90404 USA

**Keywords:** Palbociclib, Alpelisib, Translational

## Abstract

**Background:**

Combined targeting of CDK4/6 and ER is now the standard of care for patients with advanced ER+/HER2− breast cancer. However, acquired resistance to these therapies frequently leads to disease progression. As such, it is critical to identify the mechanisms by which resistance to CDK4/6-based therapies is acquired and also identify therapeutic strategies to overcome resistance.

**Methods:**

In this study, we developed and characterized multiple in vitro and in vivo models of acquired resistance to CDK4/6-based therapies. Resistant models were screened by reverse phase protein array (RPPA) for cell signaling changes that are activated in resistance.

**Results:**

We show that either a direct loss of Rb or loss of dependence on Rb signaling confers cross-resistance to inhibitors of CDK4/6, while PI3K/mTOR signaling remains activated. Treatment with the p110α-selective PI3K inhibitor, alpelisib (BYL719), completely blocked the progression of acquired CDK4/6 inhibitor-resistant xenografts in the absence of continued CDK4/6 inhibitor treatment in models of both *PIK3CA* mutant and wild-type ER+/HER2− breast cancer. Triple combination therapy against PI3K:CDK4/6:ER prevented and/or delayed the onset of resistance in treatment-naive ER+/HER2− breast cancer models.

**Conclusions:**

These data support the clinical investigation of p110α-selective inhibitors of PI3K, such as alpelisib, in patients with ER+/HER2− breast cancer who have progressed on CDK4/6:ER-based therapies. Our data also support the investigation of PI3K:CDK4/6:ER triple combination therapy to prevent the onset of resistance to the combination of endocrine therapy plus CDK4/6 inhibition.

## Background

Targeting CDK4/6 signaling in combination with endocrine therapies significantly improves progression-free survival (PFS) overall survival [1] in patients with advanced estrogen receptor-positive /HER2-negative (ER+/HER2−) breast cancer [2–4] and is now the standard of care for this disease. Three CDK4/6 inhibitors are now approved for the treatment of ER+/HER2− metastatic breast cancer: palbociclib (Ibrance®), ribociclib (Kisqali®), and abemaciclib (Verzenio®). However, despite the clinical advances associated with the addition of CDK4/6 inhibitors to endocrine therapies, acquired resistance to approved treatments for ER+/HER2− breast cancer remains a significant unmet clinical need, particularly in the metastatic setting.

Preclinical work in our laboratory first demonstrated that hormone receptor-positive breast cancer cell lines are differentially sensitive to the CDK4/6 inhibitor palbociclib when compared to other breast cancer subgroups [5]. Clinical translation of these data ultimately led to the subsequent approval of palbociclib for the treatment of advanced breast cancer in combination with hormonal targeted therapy [5, 6]. Multiple lines of preclinical evidence also support the interplay between ER and CDK4/6 signaling. Mitogenic action of estrogen in ER-dependent breast cancers is mediated via the induction of Cyclin D1 that can then bind to CDK4 and CDK6 resulting in the hyperphosphorylation of the retinoblastoma (Rb) tumor suppressor protein [7]. This in turn leads to cell cycle progression from G1 to S phase and subsequent cell proliferation [8].

Although ER positivity is a predictive biomarker of response to CDK4/6-based therapies, factors associated with the acquisition of resistance are still poorly understood. Preclinical and clinical studies have implicated a number of molecular alterations that either directly activate CDK4/6 signaling or activate escape signaling pathways in acquired CDK4/6 inhibitor resistance. These include but are not limited to Cyclin E amplification and/or Rb loss [9–11], CDK6 amplification [12], deregulated Hippo signaling [13], amplification and aberrant activity of FGFR [14], MAPK pathway activation [15], compensatory PI3K-dependent activation of non-canonical Cyclin D1-CDK2 [9], and alterations in PDK1 and PI3K/AKT signaling [16]. The phosphatidylinositol 3-kinase/AKT/mammalian target of the rapamycin (PI3K/AKT/mTOR) pathway is a key signaling driver of cellular proliferation and survival of cancer cells. Dysregulation and activation of this pathway can drive tumorigenesis of ER+/HER2− breast cancers and is associated with resistance to anti-estrogen-targeted therapies [17, 18]. Previous studies in our laboratory have demonstrated that inhibitors of the PI3K/AKT/mTOR pathway have selective activity in ER+ breast cancer cell lines carrying activating mutations in *PIK3CA* [19, 20]. In the SOLAR-1 Phase III clinical trial, patients with *PIK3CA*-mutated ER+/HER2− breast cancer had almost a doubling in PFS in response to the p110α-selective PI3K inhibitor alpelisib (BYL719) (Piqray®) in combination with fulvestrant compared to patients treated with fulvestrant with placebo [21]. Collectively, these data suggest a complex interplay between PI3K, ER, and CDK4/6 signaling that may drive ER+/HER2− breast cancers to respond and then progress through currently approved therapies.

In this study, we used in vitro and in vivo breast cancer models of acquired resistance to CDK4/6-based therapies to characterize molecular mechanisms associated with therapeutic resistance. We assessed the potential of pharmacologically targeting PI3K, ER, or CDK4/6 signaling to prevent or reverse acquired resistance. These data provide insight into the design of optimal therapeutic strategies to overcome therapeutic resistance in ER+/HER2− breast cancer.

## Methods

### Cell lines, cell culture, and reagents

Human breast cancer cell lines were maintained in appropriate culture media (e.g., RPMI 1640, DMEM, L-15) supplemented with 10 to 15% heat-inactivated fetal bovine serum (FBS), 2 mmol/L glutamine, and 1% penicillin G-streptomycin-fungizone solution (PSF, Irvine Scientific) as previously described [22]. Cells were routinely assessed for mycoplasma contamination using a multiplex PCR method, and STR profiling by the GenePrint 10 System (Promega) was used for cell line authentication. Ribociclib (LEE011-succinate), alpelisib (BYL719), and everolimus (RAD001) were provided by Novartis. Palbociclib (PD-0332991-HCL) and abemaciclib (LY2835219-mesylate salt) were purchased from Selleck Chemicals (Houston, TX). The palbociclib-resistant EFM19 (EFM19-PR) breast cancer cell line was established through long-term culture in the presence of increasing concentrations (10–1000 nmol/L) of palbociclib. For molecular analysis, PR cells were removed from the drug for 7 days prior to experiments.

### In vitro proliferation assays

Cells were seeded in 48-well plates at a seeding density optimized to maximize growth over a 6-day treatment window. After 24 h, the cells were treated with six 1:10 serial dilutions of inhibitor starting at 10 μmol/L. Control wells were imaged at this time to establish baseline cell numbers. Six days post-treatment, cells were then counted on a custom automation platform designed by Tecan (Männedorf, Switzerland). This robotic system trypsinizes adherent cells, centrifuges cells into the plane at the bottom of the wells, and counts them via brightfield image segmentation on a Synentec Cellavista® (Elmshorn, Germany) imaging system. Data are presented as % inhibition of cell generation/doubling time; inhibition of > 100% is considered to be the induction of lethality; that is, the number of cells at day 6 was less than the number of cells at day 1. Drug combination studies were carried out as described above with fixed molar ratios of drugs prepared before drug exposure on day 1.

### Western blotting and RPPA analyses

Protein lysates were obtained from cells or from snap-frozen xenograft tissue excised between 2 and 4 h post-final treatment (indicated in the figure legends). Samples were lysed using a lysis buffer (Cell Signaling Technology) containing a mixture of protease inhibitors (Calbiochem) and 1 mmol/L phenylmethylsulfonylfluoride. Western blot analysis was performed as previously described [20]. Baseline total and phosphoprotein levels of a list of > 280 protein analytes enriched for proteins known to be involved in cancer biology were determined using the reverse phase protein array (RPPA) from the core service facility at MD Anderson Cancer Center. Cell preparation and analyses were performed in accordance with MD Anderson’s published protocols. Normalized, median-centered log (expression) values were provided for all samples. Alterations in protein expression between resistant and parental cell lines, and/or following drug-treatment, were calculated as the fold change in the expression compared to control (parental baseline—or untreated/vehicle-treated control samples). For cell line studies, palbociclib-resistant (PR) cells were removed from the drug for 7 days prior to experiments. For xenograft studies, mean expression log2 expression values were calculated from replicate animals within each treatment group. Proteins measured by mouse antibodies were removed from the analysis in order to avoid contamination with background mouse/host signal. Significantly altered proteins (> 0.25; <− 0.25 log2 fold change) are depicted in heatmaps and subjected to pathway analysis using the Enrichr software (http://amp.pharm.mssm.edu/Enrichr/) [23].

### Xenograft efficacy studies

Xenograft models of ER+/HER2− breast cancer cell lines and patient-derived xenografts (PDX) were established in 6-week-old CD-1 athymic nude mice (Charles River Laboratories). For cell line xenograft studies, 17-β-estradiol 60-day release pellets (Innovative Research of America) were implanted subcutaneously into the left flank 7 days before inoculation with cells. When tumors reached an average size of 150–300 mm^3^, mice were randomized into treatment groups. Tumor xenografts were measured with calipers 3 times/week, and tumor volume in cubic millimeter was determined by multiplying height × width × length. Data were analyzed using the StudyLog® Software (San Francisco, CA). For cell line xenograft studies, fulvestrant (Faslodex, AstraZeneca) was purchased from the UCLA pharmacy. Studies with the HBCx-34 ER+/HER2− breast cancer PDX model were carried out at XenTech (Evry, France) [24]. For this study, supplementary estrogen was supplied via drinking water (β-estradiol, 8.5 mg/L), from the date of tumor implant to the date of initiation of treatment (10 mice per group). Letrozole (Femara®) was supplied by Novartis. All animal work was carried out under a protocol approved by IACUC and the UCLA Animal Research Committee. Statistical differences between treatment arms at specific time points were performed using a two-tailed paired Student *t* test (Supplemental Tables S6-S18). Differences between the groups were considered statistically significant at *P* < 0.05. All statistics were calculated using Microsoft Excel.

### Development of acquired therapy-resistant models in vivo

The ER+/HER2− breast cancer cell lines, MCF7 and HCC1500, were established as xenografts in CD-1 athymic nude mice as described above. For each cell line model, mice were treated with either vehicle control or 50–100 mg/kg palbociclib daily until xenografts began to progress on treatment. Xenografts, progressing on treatment, were serially passaged into new animals and allowed to establish in mice (3–4 weeks) prior to the re-introduction of palbociclib treatment. Once palbociclib resistance was confirmed in second-generation mice, combination treatment with fulvestrant (5 mg per mouse once weekly) was introduced. After sufficient numbers of palbociclib/fulvestrant-resistant xenografts were established, mice were then randomized into new treatment arms. Tumor pieces from resistant xenografts were also cryopreserved for use in future studies. Cell line identity for xenografts was confirmed by STR profiling between passages.

Ribociclib/fulvestrant-resistant xenografts were established through a long-term treatment of mice bearing HCC1500 xenografts with 75 mg/kg ribociclib daily plus 5 mg per mouse fulvestrant once weekly. Xenografts progressing on ribociclib/fulvestrant after 6 weeks of treatment were randomized into new treatment groups. Alpelisib/fulvestrant-resistant xenografts were generated through a long-term treatment of mice bearing ZR751 xenografts with 35 mg/kg alpelisib plus 5 mg/mouse fulvestrant once weekly.

## Results

### Acquired resistance to CDK4/6 inhibitor monotherapy is associated with loss of dependence on pRb and activation of PI3K/mTOR signaling

**Fig. 1:**
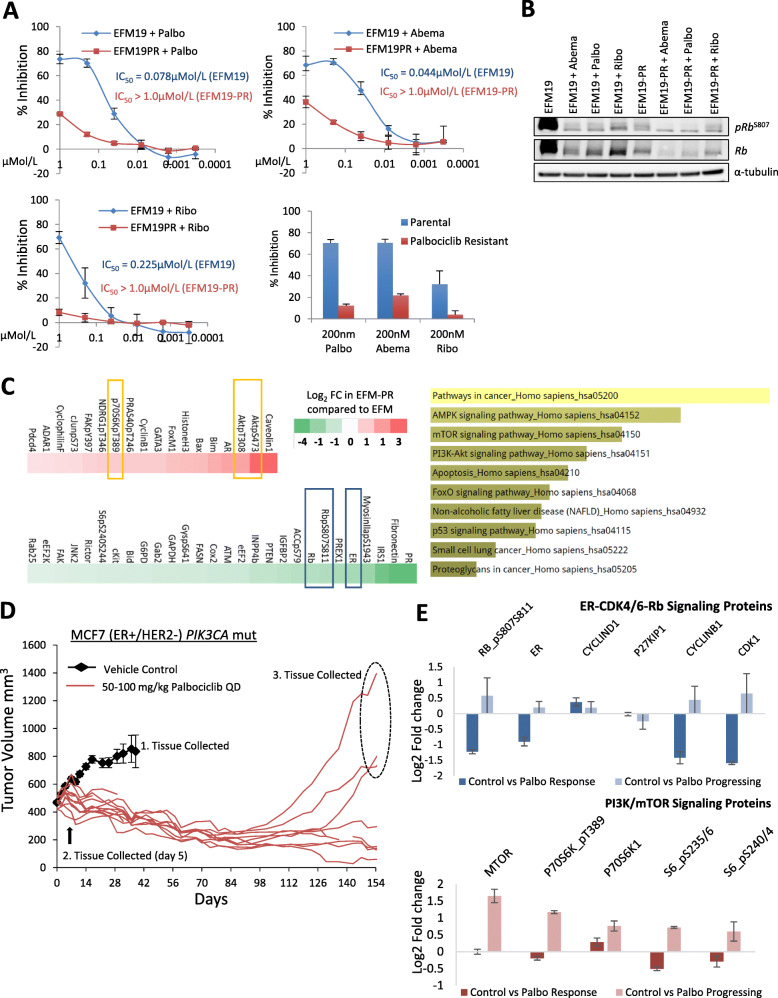
Acquired resistance to palbociclib confers resistance to ribociclib and abemaciclib and is associated with activation of the PI3K signaling pathway. **a** Effect of palbociclib, abemaciclib, and ribociclib on EFM19 and palbociclib-resistant EFM19 (EFM19-PR) cells. Bar chart, relative % growth inhibition at a concentration > EFM19 IC_50_ for each molecule, 200 nM. **b** Effect of single-dose CDK4/6 inhibitor treatment (200 nmol/L) on Rb signaling. Resistant cells cultured in the absence of palbociclib for > 7 days prior to assay. **c** Heatmap of proteins altered by > 0.25 or < − 0.25 log2 fold in EFM19-PR-resistant cells compared to EFM19 parental cells (both grown in the absence of drug) by RPPA analysis. Yellow bars highlight the PI3K/mTOR-associated proteins, and blue bars indicate the ER-CDK4/6-Rb-associated proteins. Bar graph depicts the top 10 enriched pathways (Kegg 2016; Enrichr) whereby the size of the bar chart indicates the strength of the association with each pathway. **d** MCF7 (ER+/HER2−) breast cancer cell line xenografts were treated with 50–100 mg/kg palbociclib QD for over 150 days. Xenograft tissue collected snap frozen at time points indicated. **e** Fold changes in protein levels in response to palbociclib treatment or acquirement of palbociclib resistance, as determined by RPPA analysis. Error bars represent ± SEM

In order to assess the molecular alterations directly associated with the acquirement of resistance to CDK4/6 therapy, we developed in vitro and in vivo models of acquired resistance to palbociclib monotherapy. The EFM19 (ER+/HER2−; *PIK3CA* mt) breast cancer cell line was conditioned through long-term exposure to increasing concentrations of palbociclib until the cells continued to proliferate in the presence of drug at concentrations greater than the cellular IC_50_ (78 nmol/L) (Fig. 1a). The resulting palbociclib-resistant cell line, designated EFM19-PR, demonstrated cross-resistance to other CDK4/6 inhibitors, abemaciclib and ribociclib (Fig. 1a). Previous published work from our laboratory has shown that palbociclib and abemaciclib have more potent anti-proliferative activity in ER+ breast cancer cell lines among a broad panel of human breast cancer cells [5, 22]. Here, we confirmed that ribociclib (LEE011) has the same selective activity in ER+ breast cancer cell lines (Supplemental Figure S1 and Supplemental Table S1).

Response of the parental EFM19 cells to palbociclib, abemaciclib, and ribociclib treatment was marked by an on-target reduction in total and phosphorylated Rb (pRb^S807^) (Fig. 1b). In contrast, the EFM19-PR cells showed a loss of both total and pRb at baseline, despite growing in the absence of palbociclib for 7 days, indicating loss of dependence on CDK4/6 signaling in the resistance setting (Fig. 1b). In-depth profiling of the acquired resistant cells by reverse phase protein array (RPPA) analysis identified that loss of phosphorylated pRb^S807/S811^ and total Rb protein was accompanied by a significant decrease in ER-α protein levels, indicating a loss of dependency on both ER and CDK4/6-Rb signaling in the acquired resistant cells (Fig. 1c, Table S2). Additionally, proteins associated with activated PI3K-AKT-mTOR signaling (i.e., upregulation of pAKT^S473^, pAKT^Th308^, P70S6K and downregulation of PTEN) were among those measured as significantly altered in the resistant cells relative to parental cells. In total, 25 proteins were upregulated and 18 were downregulated (> 0.25; < − 0.25 log2 fold change) in the resistant cell line compared to the parental cell line by RPPA analysis (Fig. 1c). Pathway enrichment analysis of these data identified alternations in multiple cancer-related signaling pathways including PI3K and mTOR as well as AMPK and apoptosis induction (Fig. 1c).

In a second model, MCF7 (ER+/HER2−; *PIK3CA*-mt) breast cancer cell line xenografts were conditioned through a long-term continuous treatment to progress on palbociclib monotherapy (Fig. 1d). RPPA analysis of tissue collected from xenografts responding to palbociclib (5 days of treatment) showed reduced levels of pRB^S807/811^, whereas xenografts progressing on long-term palbociclib treatment (154 days) did not show a loss of pRB^S807/811^, which is in contrast to that observed in the EFM19-PR model. A search for alterations in other proteins associated with ER-CDK4/6-Rb signaling, as measured by RPPA, found no significant changes in ER-α or Cyclin D1 protein levels; however, significant loss of the CDK2/CyclinE1 inhibitor protein p27 was detected in the acquired resistant xenografts. Significantly elevated levels of Cyclin B and CDK1 proteins were also detected in resistance, indicating a mechanism by which these cells may have evolved to progress through mitosis (Fig. 1e, Table S3). Cyclin E1 and CDK2 proteins were not measured on the array. However, significant upregulation of proteins associated with PI3K/mTOR signaling was detected in the MCF7 xenografts progressing on palbociclib (Fig. 1e, Table S3). These data suggest that despite the multiple mechanisms by which ER+/HER2− breast cancer cells acquire resistance to CDK4/6 inhibition, upregulation of PI3K/mTOR signaling appears to be common, and as such may be an attractive target for reversing resistance.

### Targeting PI3K/mTOR signaling overcomes acquired resistance to CDK4/6 inhibitors

**Fig. 2:**
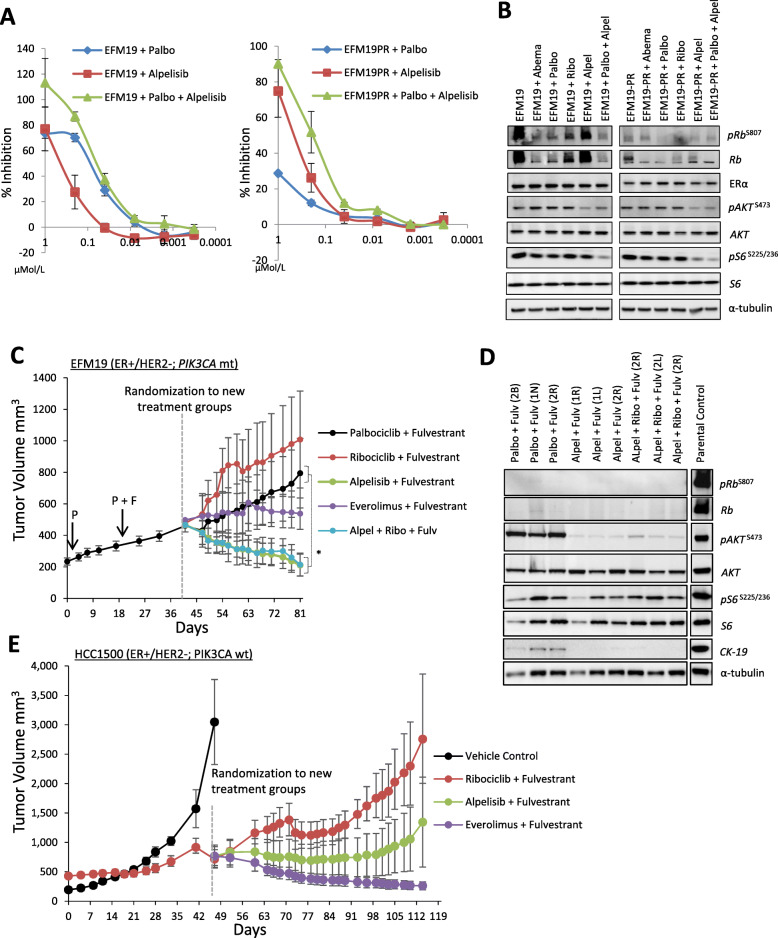
Targeting PI3K/mTOR signaling overcomes resistance to CDK4/6 inhibitors. **a** Effect of palbociclib and alpelisib alone and in combination in EFM19 and EFM-PR cells. Graphed as % inhibition of cell generation. **b** Effect of palbociclib (200 nmol/L) and aleplisib (200 nmol/L) monotherapy and combination on Rb, ER, and AKT signaling. Lysates collected 24 h post-dose. **c** Growth curves for EFM19-PR xenografts progressing on palbociclib (P)/fulvestrant (F) prior to randomization and treatment with the indicated therapeutics; 8 mice per arm (mean tumor volume ± SEM). **d** Snap-frozen xenograft tissue, collected at the end of the study from the EFM19-PR xenografts analyzed by Western blot for the indicated proteins. “Parental control” indicates xenograft tissue collected from vehicle control-treated EFM19 xenografts (Fig. 1d). CK-19 is a marker of human epithelial cell content in the snap-frozen xenograft sample; however, CK-19 expression is low in the EFM19 cells, as such α-tubulin was used as a loading control. **e** HCC1500 xenografts treated with ribociclib and fulvestrant QD until progression before re-randomization into the indicated treatment groups; 8 mice per arm (mean tumor volume ± SEM). For all experiments, the following dose schedule was used: palbociclib 100 mg/kg PO QD, ribociclib 75 mg/kg PO QD, alpelisib 35 mg/kg PO QD, and fulvestrant 5 mg/mouse QW by subcutaneous injection. *Statistically significant difference compared to control (palbociclib + fulvestrant); *P* < 0.05

To determine if inhibitors of the PI3K/mTOR signaling pathway could overcome acquired CDK4/6 inhibitor resistance, we performed in vitro and in vivo drug combination assays using selective inhibitors of PI3K/mTOR signaling. EFM19 and EFM19-PR cells show comparable sensitivity in vitro to alpelisib (p110α-selective PI3K inhibitor) single agent and combination with palbociclib (Fig. 2a). Combined palbociclib/alpelisib treatment effectively blocked the phosphorylation of Rb, AKT, and S6 in the EFM19 cells (Fig. 2b). pRb^S807^ levels were lost at baseline in palbociclib-resistant EFM19-PR cells, as such treatment with inhibitors of CDK4/6 had minimal impact on pRb^S807^ signaling. However, treatment with alpelisib monotherapy was effective in reducing both pAKT^S473^ and pS6^S235/236^ levels in the EFM19-PR cells (Fig. 2b). Taken together, these data indicate that in the acquired resistance setting, palbociclib is no longer effective in blocking cell growth due to a loss of dependence on Rb signaling; however, blocking AKT/S6 signaling by targeting PI3K is effective in blocking proliferation of palbociclib-resistant cells.

Xenografts of the EFM19-PR cell line, subcutaneously implanted into CD-1 nude mice, maintained resistance to palbociclib in vivo (Supplemental Figure S2). Furthermore, the addition of fulvestrant to palbociclib therapy did not impact the growth rate of these xenografts, indicating that acquired resistance to CDK4/6 inhibition also confers resistance/reduced sensitivity to ER signaling blockade (Fig. 2c). Xenografts progressing on palbociclib/fulvestrant combination therapy were then randomized into new treatment groups to receive either an alternative CDK4/6 inhibitor or an inhibitor of PI3K/mTOR pathway signaling in combination with fulvestrant (Fig. 2c). Animals that were switched to another CDK4/6 inhibitor (ribociclib) plus fulvestrant continued to progress through treatment. However, when the CDK4/6 inhibitor was replaced by a PI3K inhibitor (alpelisib), uniform xenograft regressions were observed (Fig. 2c). Switching from palbociclib/fulvestrant to everolimus/fulvestrant also showed modest tumor growth inhibition (Fig. 2c). The addition of ribociclib to the doublet combination of alpelisib and fulvestrant did not further enhance observed tumor regressions of the doublet. (Fig. 2c). Treatment with alpelisib as either a doublet with fulvestrant or a triplet with fulvestrant plus ribociclib effectively blocked AKT signaling in xenograft tissues (Fig. 2d).

A second resistance model was set up to determine if targeting PI3K/mTOR could reverse resistance to CDK4/6-based therapy in *PIK3CA* wild-type xenografts. Mice, bearing HCC1500 (ER+/HER2−; *PIK3CA*-wt) cell line xenografts initially responded to ribociclib/fulvestrant combination therapy before eventually progressing on treatment after 3 weeks of dosing (Fig. 2e). Mice with xenografts progressing on ribociclib/fulvestrant were then randomized to new treatment groups to either continue on ribociclib/fulvestrant or be switched to alpelisib/fulvestrant or everolimus/fulvestrant. Switching to alpelisib- or everolimus-based therapies immediately halted xenograft progression (Fig. 2e). The fact that these xenografts lack an activating mutation in *PIK3CA*, but still exhibited a significant growth inhibitory impact from the PI3K inhibitor, is relevant. Interestingly, more sustained xenograft regressions were observed in the mice switched to mTORC1-targeted therapy compared to PI3K-targeted therapy in this *PIK3CA* wild-type model indicating that targeting mTORC1 may provide superior efficacy in the *PIK3CA* wild-type setting. In CDK4/6-resistant xenografts (either to palbociclib or ribociclib), these data show that PI3K/mTOR inhibitors can have activity.

### Targeting PI3K/mTOR signaling blocks progression on CDK4/6-endocrine-based therapy independent of CDK4/6 inhibition

**Fig. 3:**
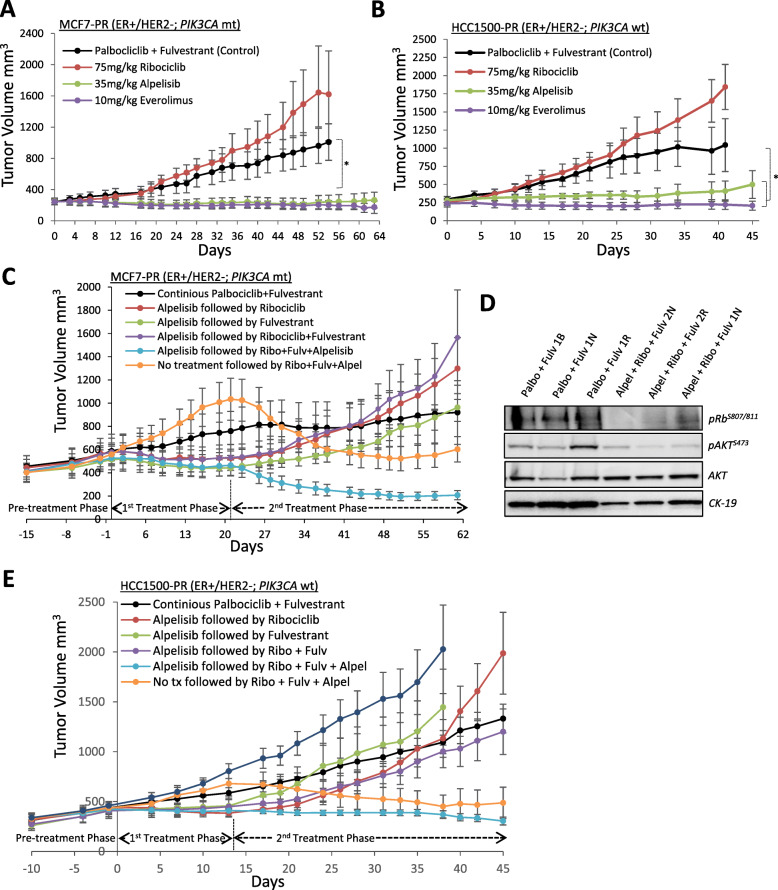
Targeting PI3K/mTOR signaling blocks the progression on CDK4/6-endocrine-based therapy in *PIK3CA* mutant and wild-type ER+/HER2− breast cancers independent of continued CDK4/6 inhibition. **a** MCF7-PR xenografts treated with ribocicllib, alpelisib, or everolimus monotherapy. **b** HCC1500-PR xenografts treated with ribocicllib, alpelisib, or everolimus monotherapy. **c** MCF7-PR xenografts progressing on palbociclib/fulvestrant were randomized into 6 treatment groups for treatment with either alpelisib alone, no treatment, or palbociclib/fulvestrant combination for 21 days prior to switching to the indicated treatments for the 2nd treatment phase; 5 mice per arm. **d** The effect of the triple combination treatment on Rb, ER, and AKT signaling measured by Western blot analysis of snap-frozen tumor tissue collected at the end of the study. CK-19 was measured as a control for human epithelial cell content. **e** HCC1500-PR xenografts progressing on palbociclib/fulvestrant before randomization treatment groups as described above for 14 days prior to switching to the indicated treatments for the 2nd treatment phase; 7 mice per arm, mean tumor volume ± SEM. For all experiments, the following dose schedule was used: palbociclib 100 mg/kg PO QD, ribociclib 75 mg/kg PO QD, alpelisib 35 mg/kg PO QD, everolimus 10 mg/kg PO QD, and fulvestrant 5 mg/mouse QW by subcutaneous injection. *Statistically significant difference compared to control (palbociclib + fulvestrant); *P* < 0.05

The potential of PI3K/mTOR monotherapy to reverse resistance to CDK4/6-based therapies, in the absence of continued CDK4/6-endocrine-based therapies, was evaluated in ER+/HER2− breast cancer cell line xenograft models conditioned to acquire resistance to palbociclib and then palbociclib/fulvestrant therapy: HCC1500 (*PIK3CA* wild-type) and MCF7 (*PIK3CA*-mutant). Mice that were continued on palbociclib/fulvestrant therapy or switched to ribociclib monotherapy showed no significant impact on xenograft tumor progression (Fig. 3a, b). However, the switch to either alpelisib or everolimus monotherapy induced complete tumor growth inhibition for over 6 weeks of treatment in both models (Fig. 3a, b).

To further investigate the potential utility of continued CDK4/6 inhibitor therapy in the acquired resistance setting, we evaluated if a CDK4/6 drug holiday (no treatment or switch to a PI3K inhibitor) could restore sensitivity to CDK4/6 inhibitors in acquired resistant models. Mice bearing MCF7-PR xenografts, progressing on palbociclib/fulvestrant, were randomized into 6 treatment groups for an initial treatment phase (Fig. 3c). Four groups were switched from the combination of palbociclib and fulvestrant to single-agent alpelisib for 21 days, and the remaining two groups of mice either remained on the combination of palbociclib and fulvestrant or given a dosing holiday for 21 days (i.e., no treatment). Significant tumor growth inhibition was observed in each of the alpelisib monotherapy-treated groups compared to both the palbociclib/fulvestrant-treated and untreated animals (Fig. 3a, 1st treatment phase). After completion of the first treatment phase, the alpelisib-treated cohorts were switched from single-agent alpelisib to either ribociclib monotherapy, fulvestrant monotherapy, ribociclib/fulvestrant combination, or triple combination of ribociclib, fulvestrant, and alpelisib. Mice switched from alpelisib to ribociclib monotherapy showed immediate xenograft progression, as did the mice switched to either fulvestrant or ribociclib/fulvestrant combination. Conversely, the triple combination of ribociclib, fulvestrant, and alpelisib resulted in robust, sustained tumor regressions that were durable throughout the 6 weeks of treatment. Even though a treatment holiday of 21 days in the first phase resulted in increased xenograft tumor growth rate, a switch to the triple combination of alpelisib/ribociclib/fulvestrant in the second phase induced tumor regressions in these very large, rapidly growing xenograft tumors (Fig. 3c). Western blot analysis of the xenograft tumor tissue showed that response to the triple combination was accompanied by a decrease in both pAKT^S473^ and pRB^S807/811^ compared to palbociclib- and fulvestrant-treated tumors (Fig. 3d).

Similar responses were observed in the *PIK3CA* wild-type HCC1500-PR model (Fig. 3e). Switch to alpelisib for 14 days inhibited xenograft tumor progression in the first treatment phase. Switching treatment back to ribociclib, fulvestrant, or the combination resulted in rapid tumor progression in the second phase. Mice given a 14-day dosing holiday were also found to be insensitive to ribociclib monotherapy. Similar to the *PIK3CA*-mutant model, robust and sustained responses were restricted to treatment arms containing the PI3K inhibitor (Fig. 3e).

In summary, the triple combination of alpelisib/ribociclib/fulvestrant induced tumor regressions in both alpelisib pretreated and untreated mice cohorts in models of acquired resistance to palbociclib/fulvestrant (Fig. 3c, e). Furthermore, single-agent treatment with either alpelisib or everolimus induced complete inhibition of tumor progression (Fig. 3a, b). These data indicate that targeting PI3K signaling, while effective on its own, does not restore sensitivity to inhibitors of CDK4/6 once resistance has been acquired. A treatment holiday is also ineffective in restoring sensitivity. AAs such, the benefit of continuing CDK4/6 based therapies in the setting of acquired resistance to inhibitors of CDK4/6 is unclear.

### Targeting CDK4/6 signaling overcomes resistance to PI3K inhibitors

**Fig. 4:**
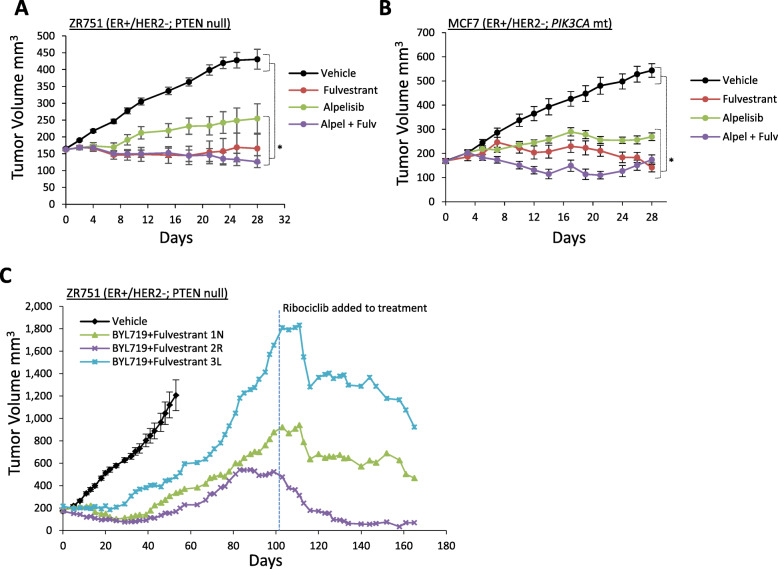
Targeting CDK4/6 overcomes acquired resistance to PI3K inhibition in ER+/HER2− breast cancer. **a**, **b** ZR751 and MCF7 xenografts treated with fulvestrant or alpelisib monotherapy or the combination (8 mice per arm, mean tumor volume ± SEM). **c** ZR751 xenografts treated with fulvestrant/alpelisib combination therapy until progression followed by the addition of ribociclib to the combination. Growth curves for 3 individual combination-treated mice are depicted. For all experiments, the following dose schedule was used: ribociclib 75 mg/kg PO QD, alpelisib 35 mg/kg PO QD, and fulvestrant 5 mg/mouse QW by subcutaneous injection. *Statistically significant difference compared to vehicle control; *P* < 0.05

To further explore the interdependency of CDK4/6 and PI3K signaling in the context of acquired resistance, we examined whether targeting CDK4/6 could overcome acquired resistance to inhibitors of PI3K. Both alpelisib and fulvestrant exhibit efficacy either in single agent or in combination in ER+/HER2− xenografts harboring *PIK3CA*-mutant (MCF7) or PTEN-null (ZR751) tumors (Fig. 4a, b). In a separate study, long-term continuous treatment (> 6 weeks) with alpelisib/fulvestrant combination therapy eventually resulted in xenograft tumor progression on treatment (Fig. 4c). Significant xenograft progression was observed for an additional 8 weeks of treatment, at which time the addition of ribociclib to the alpelisib/fulvestrant combination induced immediate regressions of these large xenografts, including one tumor that was > 1800 mm^3^ at the onset ribociclib treatment (Fig. 4c). These data demonstrate that targeting CDK4/6 can reverse acquired resistance to PI3K inhibition and provide further support for targeting the PI3K, CDK4/6, and ER signaling pathways either sequentially or as part of an upfront triple combination strategy in ER+/HER2− breast cancer.

### Upfront combined targeting of PI3K/mTOR, CDK4/6, and ER in ER+/HER2− breast cancer

**Fig. 5:**
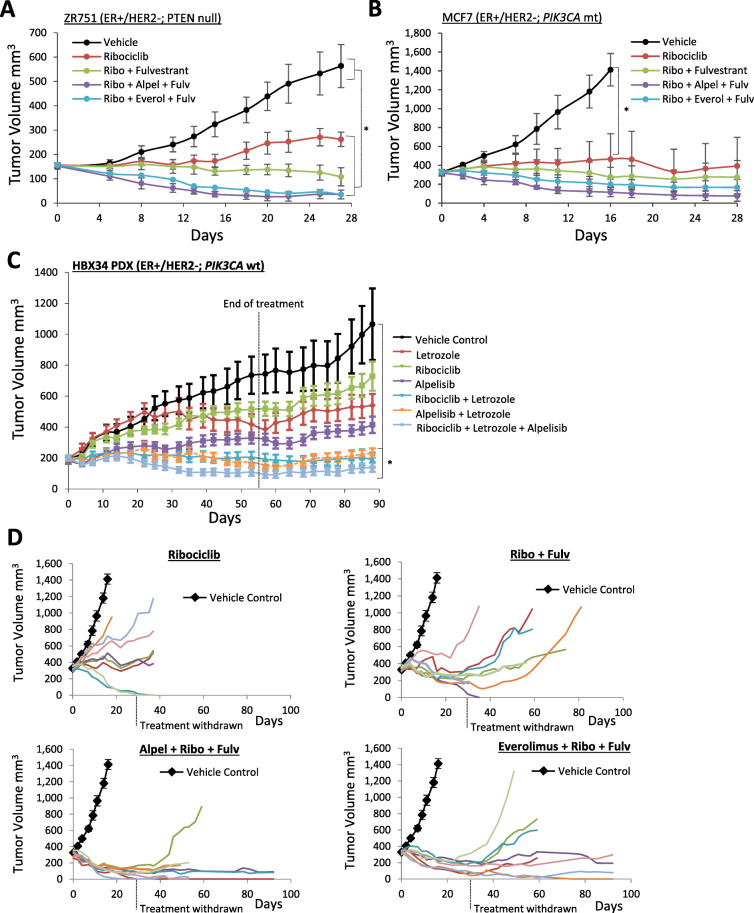
Combined targeting of ER, CDK4/6, and PI3K/mTOR in treatment-naive ER+/HER2− breast cancer xenografts. **a**, **b** ZR751 and MCF7 xenografts treated with ribociclib alone or ribociclib in combination with fulvestrant or alpelisib/fulvestrant or everolimus/fulvestrant (8 mice per arm, mean tumor volume ± SEM). **c** HBX34, a PIK3CA/PTEN wild-type PDX breast cancer model, treated with letrozole in combination with alpelisib/ribociclib. Treatment was withdrawn after 55 days, and measurements were continued for a further 40 days post-withdrawal of drug treatment. Dashed lines represent previously published data. **d** Growth curves of the individual mice (colored lines) from the MCF7 xenograft study depicted in **b** where treatment was withdrawn after 28 days and tumors were monitored for up to 9 weeks for progression. For all experiments, the following dose schedule was used: ribociclib 75 mg/kg PO QD, alpelisib 35 mg/kg PO QD, everolimus 10 mg/kg PO QD, letrozole 2.5 mg/kg PO QD, and fulvestrant 5 mg/mouse QW by subcutaneous injection. *Statistically significant difference compared to vehicle control; *P* < 0.05

The potential of simultaneously targeting ER, PI3K/mTOR, and CDK4/6 signaling de novo was evaluated in three ER+/HER2− breast cancer xenograft models representing diverse molecular backgrounds of PI3K/mTOR pathway activation. The combination of ribociclib, fulvestrant, and either alpelisib or everolimus was assessed in MCF7 (*PIK3CA*-mutant) and ZR751 (PTEN-null) xenograft models (Fig. 5a, b). The combination of alpelisib with ribociclib plus aromatase inhibitor letrozole was assessed in the HBX34 (*PIK3CA*/*PTEN* wt) patient-derived xenograft (PDX) model (Fig. 5c).

In the ZR751 and MCF7 models, the upfront triple combination with either ribociclib/fulvestrant/alpelisib or ribociclib/fulvestrant/everolimus induced significant xenograft tumor regressions, which were superior to that observed with either single-agent ribociclib or ribociclib/fulvestrant (Fig. 5a, b). Follow-up analysis, monitoring xenograft regrowth post-withdrawal of treatment, identified significantly less xenograft regrowth in the mice treated with the triplet combination of alpelisib/ribociclib/fulvestrant compared to the singlet and doublet treatment arms (Fig. 5d). At 9 weeks post-treatment, only 2 of 8 mice (25%) in the triplet arm of alpelisib/ribociclib/fulvestrant showed xenograft progression, whereas 5/7 and 6/6 xenografts in the ribociclib and ribociclib/fulvestrant arms progressed almost immediately upon withdrawal of treatment, respectively. Interestingly, significantly less xenograft regrowth was observed in the triple combination that included alpelisib compared to everolimus (Fig. 5d). Similar findings were observed in the post-treatment follow-up analysis of the ZR751 model (Supplemental Figure S3).

Superior efficacy of the triple combination was also observed in the HBX34 ER+ breast cancer PDX model that is wild type for *PIK3CA* and *PTEN*. As previously published, the combination of ribociclib and letrozole inducted greater efficacy than either single agent [24]. Here, we show that alpelisib is also combined with letrozole to induce greater anti-tumor activity. Furthermore, the triple combination of alpelisib/ribociclib/letrozole completely blocked tumor progression (Fig. 5c). Responses in the double and triple combination arms were maintained for over 4 weeks after withdrawal of treatment (Fig. 5c).

These data indicate that an upfront triple combination targeting CDK4/6, PI3K, and ER can induce complete xenograft tumor regressions and may prevent the onset of therapeutic resistance. Each of the treatment combinations was well tolerated in all models tested (Supplemental Table S4).

### Pharmacodynamic biomarkers of responses to triple combination therapy targeting PI3K, CDK4/6, and ER signaling pathways

**Fig. 6:**
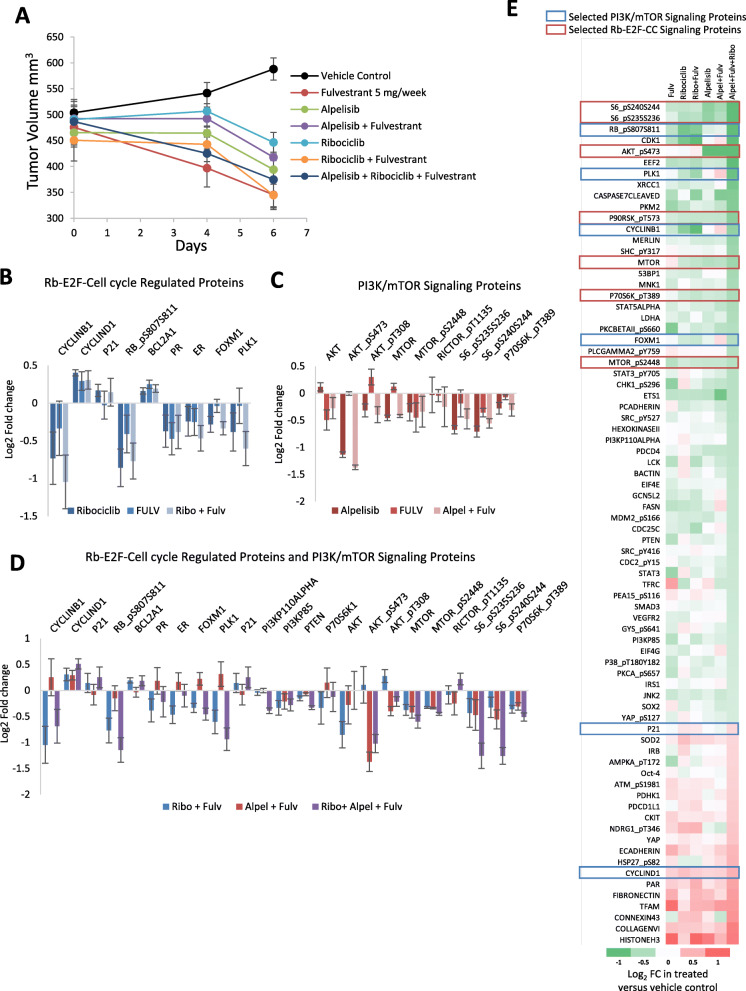
PD Biomarker analysis of responses to combined targeting of ER, CDK4/6, and PI3K/mTOR in treatment-naive ER+/HER2− breast cancer cell line xenografts. **a** MCF7 xenografts treated with ribociclib (75 mg/kg PO QD) monotherapy or combination with fulvestrant (5 mg/mouse QW), alpelisib (35 mg/kg PO QD), or everolimus (10 mg/kg PO QD) (4 mice per arm, mean tumor volume ± SEM). **b** Effect of ribociclib alone and in combination with fulvestrant on selected proteins associated with cell cycle regulation by RPPA analysis. **c** Effect of alpelisib alone and in combination with fulvestrant on selected proteins associated with PI3K/mTOR signaling. **d** Comparing the effects of the triple combination versus the double combinations on proteins selected form both cell cycle and PI3K/mTOR pathways. **e** Heatmap showing all proteins (*n* = 76) with significant fold change (< 0.25 down (*n* = 56) or < − 0.25 up (*n* = 20)) in expression in response to the triple combination treatment relative to xenografts from control-treated mice. Expression changes for the same set of proteins in response to mono or combination therapy are also shown. Error bars represent ± SEM

In order to investigate the potential mechanisms by which triple combination is effective in blocking xenograft regrowth, we performed RPPA analysis of MCF7 xenograft tissue collected from mice treated for 6 days with either single-agent, double, or triple combination regimens (Fig. 6a). RPPA analyses from 4 replicate mice in each group identified alterations of key pathway-specific signaling proteins associated with each molecule. Treatment with single-agent ribociclib and combination with fulvestrant induced on-mechanism downregulation of proteins associated with ER-CDK4/6-Rb signaling and cell cycle progression (pRb^S807/S811^, Cyclin B1, ERα, PR, FOXM1, PLK1) (Fig. 6b, Table S5), indicating target inhibition and confirming the mechanism of action of ribociclib. Xenografts from mice treated with alpelisib or combination with fulvestrant induced significant reduction in phosphorylation of proteins associated with activated PI3K/mTOR signaling (pAKT^S473^, pAKT^Th308^, pmTOR^S2448^, p70S6K^pT389^ pS6^S236/S236^, and pS6^S240/S244^) (Fig. 6c, Table S3), indicating target inhibition and confirming the mechanism of action of alpelisib. The triple combination targeting CDK4/6, PI3K, and ER appears to amplify the effect on signaling that was observed for either doublet combination (Fig. 6d, Table S5). Possible additional mechanisms by which the triple combination may be preventing the onset of therapeutic resistance were assessed by identifying proteins that were either significantly down- or upregulated (> 25%) by the triple combination (Fig. 6e). Triple combination induced very few unique changes in protein levels that were not also observed with either doublet combination (Fig. 6e). As such, it is possible that the triple combination may be preventing the onset of resistance by simply inducing greater inhibition of these targets and key signaling pathways. However, it should be noted that the RPPA platform comprised a set of proteins that are associated with known cancer-associated signaling pathways and as such provides limited scope for identifying biomarkers and/or novel signaling pathways that may be playing a role in preventing the onset of resistance. Future studies, using more comprehensive screening technologies such as RNA-seq, and using a greater number of samples, may provide more insight into the mechanisms.

## Discussion

The three approved inhibitors of cyclin-dependent kinases 4 and 6, palbociclib, ribociclib, and abemaciclib, have shown significant activity in combination with endocrine therapy in ER+/HER2− breast cancer with the largest positive impact on the outcome data in the past 45 years [2–4]. As such, they represent a new standard-of-care for this subtype of the disease. Although ER-positive status strongly predicts for response to these drugs, identifying pharmacological targets that may block the eventual onset of resistance to CDK4/6-based therapies is a critical next step. In the current study, we utilized multiple models of acquired resistance to CDK4/6-targeted therapies and confirm that cross-resistance exists among this class of compounds; however, the PI3K/mTOR pathway signaling remains activated in resistance. We show that targeting PI3K alone or in combination with inhibitors of CDK4/6 and endocrine therapies can overcome acquired resistance and potentially prevent the emergence of resistance to CDK4/6-ER-based therapies for ER+/HER2− breast cancer.

Despite the proven clinical benefit of adding CDK4/6 inhibitors to endocrine therapy for ER+/HER2− breast cancer, ultimate progression on therapy remains a significant factor impacting long-term benefits of CDK4/6-based therapies. Results from the pivotal PALOMA-2 trial, show that although the addition of palbociclib to letrozole significantly improved PFS from 14.5 to 24.8 months, progressive disease still occurs in the majority of palbociclib-treated patients [25]. The molecular mechanisms by which therapeutic resistance to CDK4/6 inhibition acquired are not well understood at present but are likely mediated by several different processes including loss of Rb protein or function; alterations in CDK4/6, Cyclin D, Cyclin E, and/or CDK repressor protein expression; activation of CDK2; or activation of PI3K signaling [11, 26, 27].

Many of the proposed resistance mechanisms such as Rb loss or CDK2 activation are not easily amenable to targeted therapeutic interventions; however, the PI3K pathway activation in breast cancer has been successfully targeted with small molecule inhibitors. Targeting downstream mTOR signaling via an mTORC1 inhibitor, everolimus, in combination with the aromatase inhibitor exemestane compared to exemestane plus placebo, improved the median progression-free survival (PFS) in ER+/HER2− advanced breast cancer from 3.2 to 7.8 months (hazard ratio 0.38 [95% CI 0.31–0.48], *P* < 0.0001) [28]. Targeting the PI3K pathway upstream at AKT has had limited efficacy in breast cancers as shown with the selective AKT1/2 inhibitor, ipatasertib [29]. The majority of the clinical investigation associated with targeting the PI3K/AKT/mTOR pathway, upstream of mTORC1, has focused on directly targeting the catalytic subunit of the PI3K enzyme that is frequently activated through somatic mutation of the *PIK3CA* gene [30]. Initial attempts to target the PI3K enzyme complex in ER+/HER2− breast cancer using “pan-PI3K inhibitors” that inhibit all four isoforms (α, β, γ, and δ) of the class IA PI3Ks were limited by toxicity [31, 32]. Conversely, a significantly greater promise has been observed in ER+/HER2− breast cancers using an α-selective molecule. Combination of the p110α-selective PI3K inhibitor BYL719 (alpelisib; Novartis) with fulvestrant in patients with ER+/HER2− metastatic breast cancer carrying activating *PIK3CA* mutations that had progressed on a previous endocrine therapy induced an improvement in PFS of 11.0 months compared to 5.7 months in the placebo plus fulvestrant group (hazard ratio 0.65, 95% CI 0.50 to 1.25, *P* = 0.00065) [21]. These data support the hypothesis that *PIK3CA* mutations can drive the progression of a subtype of ER+/HER2− breast cancers, including patients whose disease progressed on hormone therapy. Given that the standard-of-care for many ER+/HER2− breast cancers is now hormone therapy in combination with a CDK4/6 inhibitor, there is a strong rationale to suggest inhibitors of PI3K/mTOR signaling may also be active in this arena.

In the present study, we generated in vitro and in vivo models of ER+/HER2− breast cancer with acquired resistance to both CDK4/6 inhibitor monotherapy and combination therapy with hormone blockade. Acquired resistance to inhibitors of CDK4/6 is characterized by a loss of dependence on ER-CDK4/6-Rb signaling, as demonstrated by cross-resistance to other CDK4/6 inhibitors in this class in both in vitro and in vivo assays. Proteomic profiling of the acquired resistant cell lines indicates that loss of dependence on ER-CDK4/6-Rb signaling in our models can occur either through direct loss of total and phosphorylated Rb protein or a loss of dependence on Rb by a loss of a negative regulator protein such as p27, which can lead to activated Cyclin E1-CDK2 signaling [33, 34]. Other preclinical studies have shown that both Rb protein and its transcript are lost in acquired resistance to palbociclib [9, 35]. Collectively, these data indicate that switching patients with disease progression on palbociclib therapy to another CDK4/6 inhibitor is unlikely to provide benefit. These findings are particularly important given that there are currently three clinically approved CDK4/6 inhibitors; loss of dependence on ER-CDK4/6-Rb signaling would confer cross-resistance of each of these compounds. However, significantly upregulated PI3K-AKT-mTOR signaling was found to be common in both models with acquired resistance to palbociclib that were assessed by RPPA analysis, indicating a potential role as an escape signaling pathway for PI3K/AKT/mTOR signaling in acquired resistance to CDK4/6 inhibitors. These data coupled with the recent clinical data emerging around p110α-selective molecules in ER+/HER2− breast cancer indicate that exploring PI3K inhibition as a means to overcome and/or prevent resistance to CDK4/6-based therapies could be a very attractive approach that might be fast-tracked towards clinical translation.

The data presented in this study show that pharmacologically targeting PI3K/mTOR signaling inhibits the growth of cell lines and xenografts conditioned to progress on CDK4/6-based therapies. ER+/HER2− breast cancer xenografts, progressing on palbociclib/fulvestrant, were unresponsive to either an immediate switch to an alternative CDK4/6 inhibitor or a switch following a dosing holiday. The rate of tumor progression in each of these acquired resistance models could only be impacted by the inclusion of a PI3K/mTOR pathway inhibitor in the treatment schedule. The addition of alpelisib to ribociclib/fulvestrant induced significant xenograft regressions in two separate models tested. Similar efficacies were also observed with the alpelisib/fulvestrant doublet combination in the absence of continued CDK4/6 inhibitor treatment, underscoring the importance of the PI3K inhibition. Moreover, monotherapy with either alpelisib or everolimus completely blocked xenograft growth in both *PIK3CA* mutant and wild-type models. These data provide further evidence that continued treatment with inhibitors of CDK4/6 is unlikely to provide benefit either a single-agent or as a combination partner post-progression on CDK4/6-based therapies.

Targeting PI3K in CDK4/6-resistant breast cancer cells appears to be effective in both *PIK3CA* mutant and wild-type models, whereas targeting mTORC1 is effective in *PIK3CA* wild-type xenograft and one of two *PIK3CA*-mutant models. Additional preclinical and clinical studies are required to investigate this observation. It is likely that targeting PI3K/mTOR signaling at different nodes in the pathway may have different affects on tumor progression depending on the driving alteration. Although our data show that targeting PI3K is likely to benefit patients with *PIK3CA*-mutant ER+/HER2− breast cancer that has progressed on CDK4/6-targeted therapy, targeting mTORC1 may provide another therapeutic option for patients with *PIK3CA* wild-type breast cancer. Previous studies have shown that activated PI3K/mTOR signaling may be playing a role in resistance to CDK4/6-based therapies in ER+ breast cancer [9, 36]. Although it has been reported that targeting PI3K may be ineffective once acquired resistance to CDK4/6 inhibitors occurs [9], we found that PI3K inhibition can reverse resistance in multiple models of acquired CDK4/6 inhibitor resistance. It is possible that differences observed in the present study may be due to the use of the p110α-selective inhibitor alpelisib, over a pan-PI3K inhibitor. Additional evidence for a strong interplay between the CDK4/6 and PI3K pathways is provided by our observation that ER+/HER2− xenografts progressing on an alpelisib/fulvestrant combination could be effectively reversed by the addition of the CDK4/6 inhibitor, ribociclib. These data are consistent with previous reports of in vitro studies that show CDK4/6 inhibitors can reverse acquired resistance to inhibitors of PI3K [36]. Data from two clinical studies with small numbers of patients indicate that palbociclib-based therapies have limited efficacy after progression on the mTORC1 inhibitor everolimus (RAD001) [37, 38]. However, the efficacy of CDK4/6 inhibition post-progression on PI3K inhibitor-based therapies is yet to be investigated.

Although our data suggest that the benefit of continued use of selective inhibitors of CDK4/6 in the acquired resistant setting will be of little or no clinical benefit, combined targeting of CDK4/6 and PI3K/mTOR signaling with hormonal blockade may provide benefit to treatment-naive ER+/HER2− breast cancers. Here, we report that upfront triple combination therapy can prevent/delay the onset of resistance in xenograft models. Sustained tumor regressions were observed for over 9 weeks post-drug withdrawal in xenografts treated with CDK4/6:PI3K:ER combination therapy. Similar responses were observed in a PDX model of ER+/HER2− breast cancer when fulvestrant was replaced by the aromatase inhibitor, letrozole. These data indicate that targeting CDK4/6 and PI3K may be effective in combination with multiple classes of endocrine-based therapies. Moreover, we have shown that triple combination therapy is effective in both “PI3K-activated” (*PIK3CA*-mutated or PTEN-null) and “PI3K-normal” (*PIK3CA/PTEN* wild-type) ER+/HER2− breast cancer xenografts. The data presented here indicate that the mechanisms by which the triple combination blocks xenograft regrowth are through enhanced inhibition of both PI3K/AKT/mTOR and CDK4/6-Rb/ERα signaling, as opposed to the combination hitting a novel target/signaling pathway. It is possible that this simultaneous inhibition on each pathway leads to the complete arrest of cell cycle progression and ultimate induction of apoptosis. Although long-term treatment with this triple combination was well tolerated in mice, careful dose management of the combination strategy will be required in human studies. Encouraging data have been reported from phase I/II clinical studies investigating triplet CDK4/6:PI3K-mTOR:ER combination strategies. Clinical activity and an acceptable safety profile were observed in response to triple combination treatment with ribociclib (3 weeks on, 1 week off) plus alpelisib (continuous) and letrozole (continuous) in heavily pretreated ER+/HER2− breast cancer patients [39]. Continuous treatment of triple combination therapy with ribociclib/everolimus/exemestane has also been shown to be well tolerated and demonstrate clear clinical benefit in patients with advanced ER+/HER2− breast cancer [40].

## Conclusions

The preclinical data presented here strongly support the clinical testing of PI3K/mTOR pathway inhibitors, in particular p110α-selective PI3K inhibitors such as alpelisib, in patients with ER+/HER2−breast cancer experiencing disease progression on CDK4/6-based therapies. Our data also support the inclusion of *PIK3CA* wild-type patients in these studies. Recent clinical data showing that alpelisib can be combined effectively and safely with hormone therapy in patients with ER+/HER2− breast cancer provide additional support for this approach. Finally, our data also support the evaluation of upfront triple PI3K:CDK4/6:ER combination therapy to delay and/or prevent the acquisition of therapeutic resistance in ER+/HER2− breast cancer.

## Supplementary information

**Additional file 1: Figure S1.** Activity of ribociclib (NVP-LEE011) in breast cancer cell lines. In vitro IC_50_s (generational inhibition) for each of the breast cell lines. Data represent mean IC50 +/- 95% confidence interval where available. Hormone receptor positive (ER+) cell lines highlighted in yellow. All experiments were repeated in at least duplicate.

**Additional file 2: Figure S2.** EFM19-PR cell line xenografts maintain resistance to palbociclib in vivo. Growth curves for EFM19 and EFM19-PR xenografts treated with 100 mg/kg palbociclib QD, 8 mice per arm (mean tumor volume ± SEM) and waterfall plot representing the change in tumor volume after 35 days of treatment.

**Additional file 3: Figure S3.** Regrowth of xenograft tumors post withdrawal of ER, CDK4/6 and PI3K/mTOR combined targeted therapy. Growth curves of the individual mice from the MCF7 xenograft study depicted in B) where treatment was withdrawn after 28 days and tumors were monitored for up to 9 weeks for progression. For all experiments the following dose schedule was used: Ribociclib 75mg/kg PO QD, alpelisib 35mg/kg PO QD, everolimus 10mg/kg PO QD, letrozole 2.5mg/kg PO QD, fulvestrant 5 mg/mouse QW by subcutaneous injection.

**Additional file 4: Table S1.** Table listing Ribociclib IC50 in each of the breast cancer cell lines. ER, AR and HER2 levels, as measured by RPPA, also included for reference.

**Additional file 5: Table S2.** Pharmacodynamic changes in protein levels in acquired resistant cells versus parental cells. RPPA data (norm_Log2 values) restricted to the proteins with a > 0.25 or < − 0.25 difference in log 2 expression in EFM19-PR resistant cells compared to EFM19 parental cells.

**Additional file 6: Table S3.** Pharmacodynamic changes in protein levels in xenografts responding to 50-100 mg/kg palbociclib prior to progression on treatment (resistant). Individual xenograft samples either from MCF7 vehicle treated mice, palbociclib responsive mice and from mice that had progressed on palbociclib were analyzed by RPPA and the average norm_log 2 for each analyzed protein is depicted (*n* = 4 per group).

**Additional file 7: Table S4.** Mean mouse body weights in each of the experimental arms (8 mice per group). MCF7 and ZR751 combination studies, Fig. 5a and b.

**Additional file 8: Table S5.** Pharmacodynamic changes in protein levels in treated groups relative to vehicle control. As measured by RPPA. Normalized, median-centered log (expression) values were calculated for each sample, mean expression values were calculated for replicate mice within each treatment group. Alterations in protein expression between each treatment group and the vehicle control were calculated as the mean fold change in expression compared to control (parental baseline – or untreated/vehicle treated control samples).

**Additional file 9: Table S6–18.** Mean tumor volumes and mouse body weights from each of the xenograft studies presented in this manuscript.

## Data Availability

All data generated or analyzed during this study are included in this published article [and its supplementary information files].
