# Supplementary material for: Targeting activated PI3K/mTOR signaling overcomes acquired resistance to CDK4/6-based therapies in preclinical models of hormone receptor-positive breast cancer
Source: Breast Cancer Res. 2020 Aug 14;22:89. doi: 10.1186/s13058-020-01320-8 (PMC7427086; doi:10.1186/s13058-020-01320-8)
Supplement: Supplementary file 1 — Additional file 1: Figure S1. Activity of ribociclib (NVP-LEE011) in breast cancer cell lines. In vitro IC50s (generational inhibition) for each of the breast cell lines. Data represent mean IC50 +/- 95% confidence interval where available. Hormone receptor positive (ER+) cell lines highlighted in yellow. All experiments were repeated in at least duplicate. [file 13058_2020_1320_MOESM1_ESM.pptx]

## Slide 1
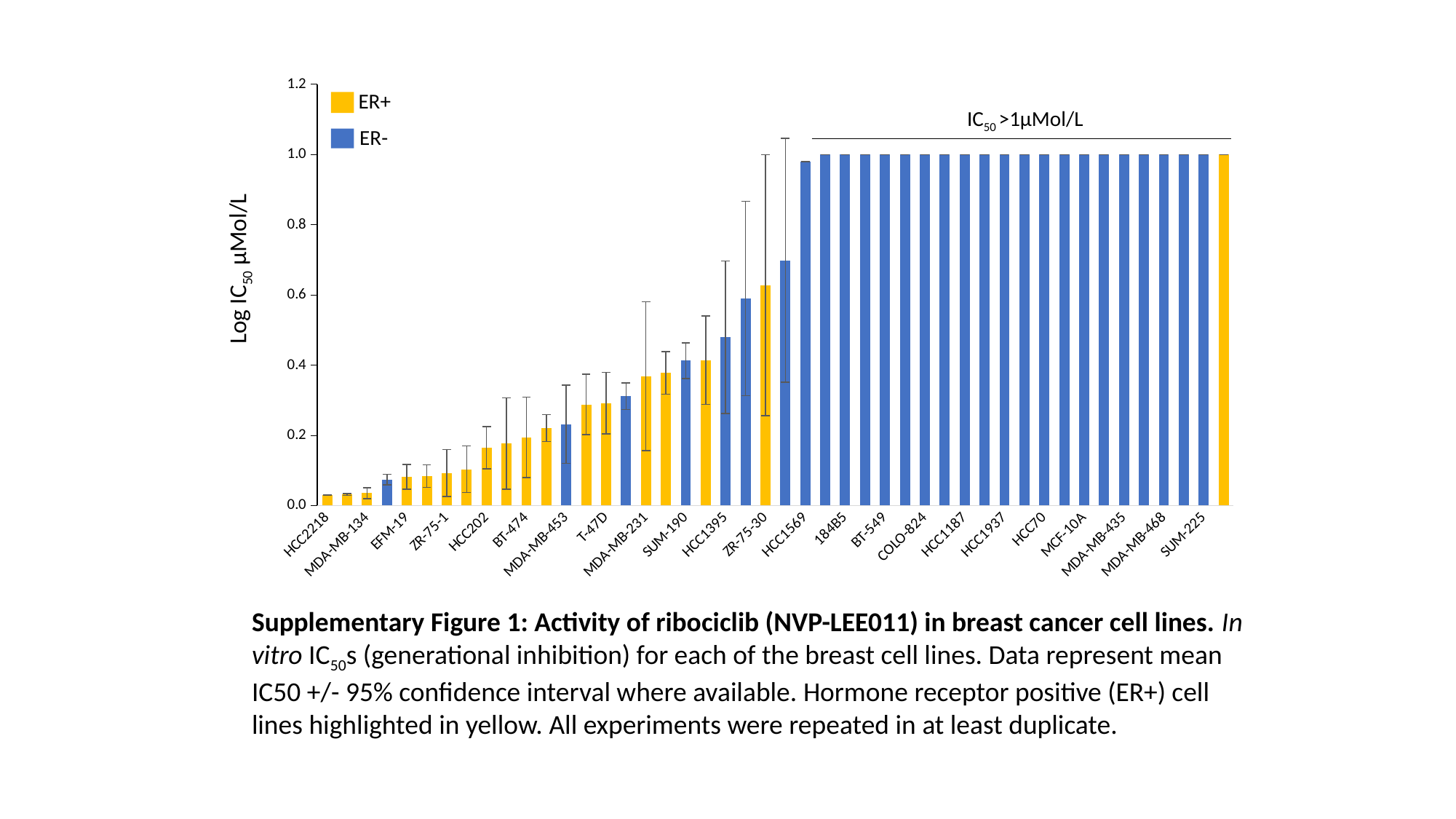

### Chart
| Category | IC50 |
|---|---|
| HCC2218 | 0.0298069999999999 |
| MDA-MB-175 | 0.0317138526357175 |
| MDA-MB-134 | 0.0354837062043976 |
| UACC-893 | 0.0740939738845205 |
| EFM-19 | 0.0818397048645132 |
| CAMA-1 | 0.0838482113583823 |
| ZR-75-1 | 0.0923023418012782 |
| MDA-MB-415 | 0.103523684939541 |
| HCC202 | 0.165238982731073 |
| HCC1419 | 0.176269275967198 |
| BT-474 | 0.194242240765036 |
| HCC1500 | 0.220753957613448 |
| MDA-MB-453 | 0.231676257976514 |
| EFM-192A | 0.288067634006243 |
| T-47D | 0.291716225722876 |
| UACC-732 | 0.311868615644473 |
| MDA-MB-231 | 0.36860964102964 |
| MCF-7 | 0.377869044184622 |
| SUM-190 | 0.412935884364631 |
| MDA-MB-361 | 0.414261814141733 |
| HCC1395 | 0.479576613720895 |
| HCC38 | 0.590143549433525 |
| ZR-75-30 | 0.627914183958286 |
| HCC1143 | 0.698744614971736 |
| HCC1569 | 0.980338 |
| 184A1 | 1.0 |
| 184B5 | 1.0 |
| BT-20 | 1.0 |
| BT-549 | 1.0 |
| CAL-51 | 1.0 |
| COLO-824 | 1.0 |
| DU4475 | 1.0 |
| HCC1187 | 1.0 |
| HCC1806 | 1.0 |
| HCC1937 | 1.0 |
| HCC1954 | 1.0 |
| HCC70 | 1.0 |
| Hs578T | 1.0 |
| MCF-10A | 1.0 |
| MDA-MB-157 | 1.0 |
| MDA-MB-435 | 1.0 |
| MDA-MB-436 | 1.0 |
| MDA-MB-468 | 1.0 |
| SK-BR-3 | 1.0 |
| SUM-225 | 1.0 |
| UACC-812 | 1.0 |ER+
ER-
IC50 >1µMol/L
Log IC50 μMol/L
Supplementary Figure 1: Activity of ribociclib (NVP-LEE011) in breast cancer cell lines. In vitro IC50s (generational inhibition) for each of the breast cell lines. Data represent mean IC50 +/- 95% confidence interval where available. Hormone receptor positive (ER+) cell lines highlighted in yellow. All experiments were repeated in at least duplicate.
