# Supplementary material for: Targeting activated PI3K/mTOR signaling overcomes acquired resistance to CDK4/6-based therapies in preclinical models of hormone receptor-positive breast cancer
Source: Breast Cancer Res. 2020 Aug 14;22:89. doi: 10.1186/s13058-020-01320-8 (PMC7427086; doi:10.1186/s13058-020-01320-8)
Supplement: Supplementary file 2 — Additional file 2: Figure S2. EFM19-PR cell line xenografts maintain resistance to palbociclib in vivo. Growth curves for EFM19 and EFM19-PR xenografts treated with 100 mg/kg palbociclib QD, 8 mice per arm (mean tumor volume ± SEM) and waterfall plot representing the change in tumor volume after 35 days of treatment. [file 13058_2020_1320_MOESM2_ESM.pptx]

## Slide 1
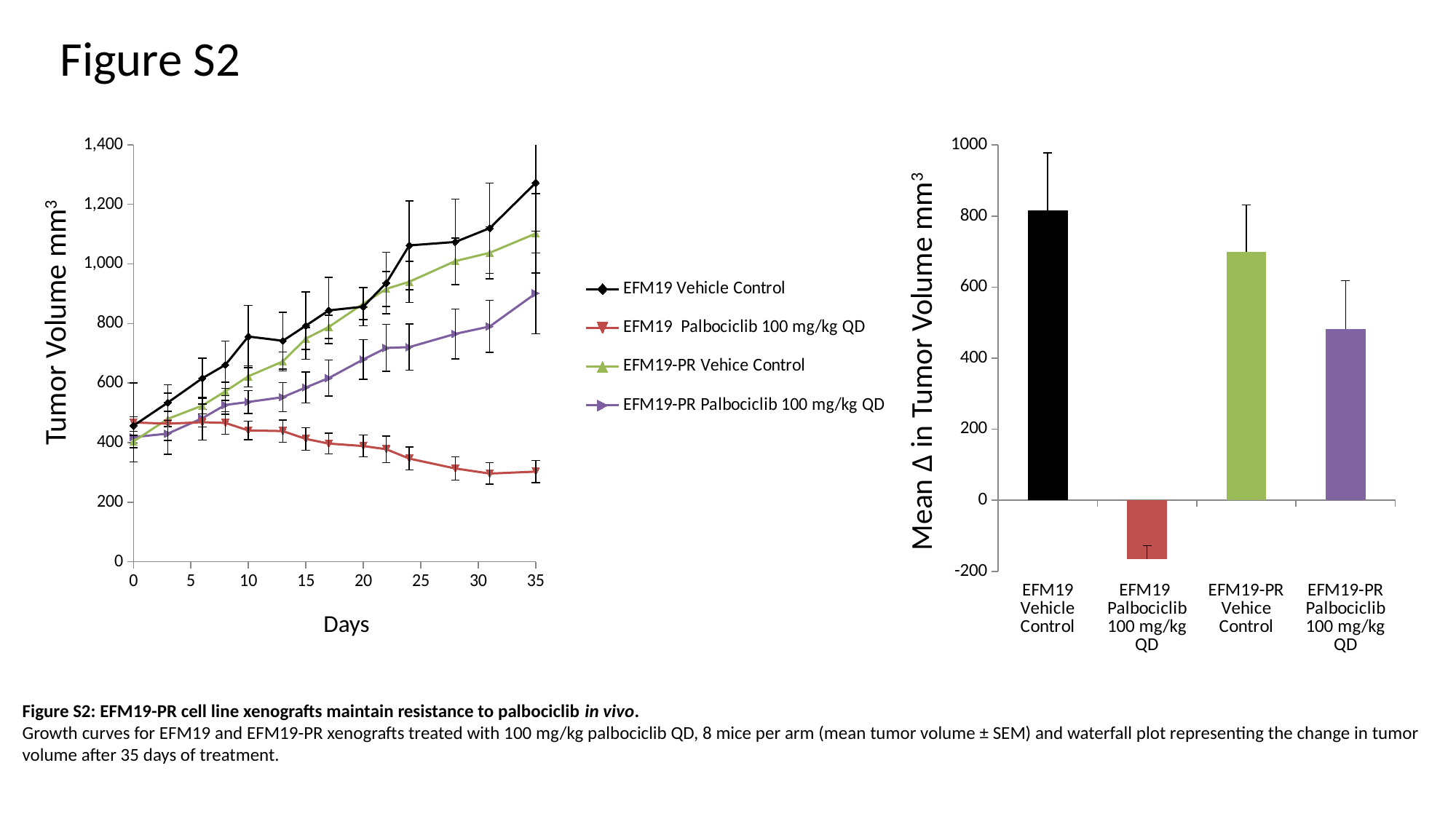

# Figure S2
### Chart
| Category | EFM19 Vehicle Control | EFM19 Palbociclib 100 mg/kg QD | EFM19-PR Vehice Control | EFM19-PR Palbociclib 100 mg/kg QD |
|---|---|---|---|---|
### Chart
| Category | |
|---|---|
| EFM19 Vehicle Control | 815.95002425 |
| EFM19 Palbociclib 100 mg/kg QD | -165.18871 |
| EFM19-PR Vehice Control | 698.4950241111112 |
| EFM19-PR Palbociclib 100 mg/kg QD | 482.9741447777781 |Tumor Volume mm3
Mean Δ in Tumor Volume mm3
Days
Figure S2: EFM19-PR cell line xenografts maintain resistance to palbociclib in vivo.
Growth curves for EFM19 and EFM19-PR xenografts treated with 100 mg/kg palbociclib QD, 8 mice per arm (mean tumor volume ± SEM) and waterfall plot representing the change in tumor volume after 35 days of treatment.
