# Supplementary material for: Targeting activated PI3K/mTOR signaling overcomes acquired resistance to CDK4/6-based therapies in preclinical models of hormone receptor-positive breast cancer
Source: Breast Cancer Res. 2020 Aug 14;22:89. doi: 10.1186/s13058-020-01320-8 (PMC7427086; doi:10.1186/s13058-020-01320-8)
Supplement: Supplementary file 3 — Additional file 3: Figure S3. Regrowth of xenograft tumors post withdrawal of ER, CDK4/6 and PI3K/mTOR combined targeted therapy. Growth curves of the individual mice from the MCF7 xenograft study depicted in B) where treatment was withdrawn after 28 days and tumors were monitored for up to 9 weeks for progression. For all experiments the following dose schedule was used: Ribociclib 75mg/kg PO QD, alpelisib 35mg/kg PO QD, everolimus 10mg/kg PO QD, letrozole 2.5mg/kg PO QD, fulvestrant 5 mg/mouse QW by subcutaneous injection. [file 13058_2020_1320_MOESM3_ESM.pptx]

## Slide 1
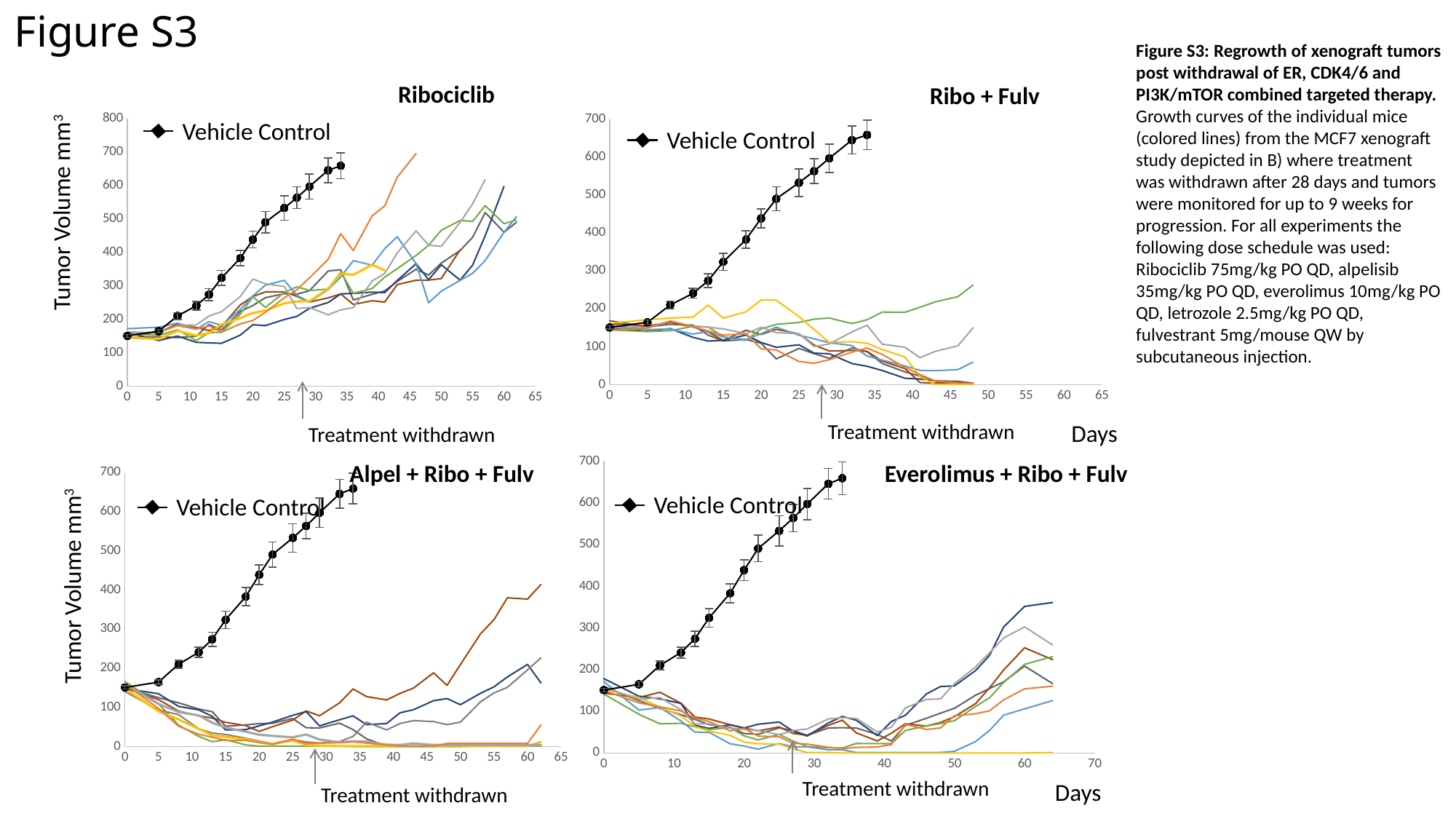

# Figure S3
Figure S3: Regrowth of xenograft tumors post withdrawal of ER, CDK4/6 and PI3K/mTOR combined targeted therapy. Growth curves of the individual mice (colored lines) from the MCF7 xenograft study depicted in B) where treatment was withdrawn after 28 days and tumors were monitored for up to 9 weeks for progression. For all experiments the following dose schedule was used: Ribociclib 75mg/kg PO QD, alpelisib 35mg/kg PO QD, everolimus 10mg/kg PO QD, letrozole 2.5mg/kg PO QD, fulvestrant 5mg/mouse QW by subcutaneous injection.
Ribociclib
Ribo + Fulv
### Chart
| Category | Vehicle Control | LEE 75 + Fulvestrant 5 mg/week | LEE 75 + Fulvestrant 5 mg/week | LEE 75 + Fulvestrant 5 mg/week | LEE 75 + Fulvestrant 5 mg/week | LEE 75 + Fulvestrant 5 mg/week | LEE 75 + Fulvestrant 5 mg/week | LEE 75 + Fulvestrant 5 mg/week | LEE 75 + Fulvestrant 5 mg/week |
|---|---|---|---|---|---|---|---|---|---|Vehicle Control
Treatment withdrawn
Days
### Chart
| Category | Vehicle Control | LEE 75mg/kg | LEE 75mg/kg | LEE 75mg/kg | LEE 75mg/kg | LEE 75mg/kg | LEE 75mg/kg | LEE 75mg/kg | LEE 75mg/kg |
|---|---|---|---|---|---|---|---|---|---|Vehicle Control
Tumor Volume mm3
Treatment withdrawn
### Chart
| Category | Vehicle Control | LEE 75mg/kg + RAD001 10 mg/kg + Fulvest 5 mg/wk | LEE 75mg/kg + RAD001 10 mg/kg + Fulvest 5 mg/wk | LEE 75mg/kg + RAD001 10 mg/kg + Fulvest 5 mg/wk | LEE 75mg/kg + RAD001 10 mg/kg + Fulvest 5 mg/wk | LEE 75mg/kg + RAD001 10 mg/kg + Fulvest 5 mg/wk | LEE 75mg/kg + RAD001 10 mg/kg + Fulvest 5 mg/wk | LEE 75mg/kg + RAD001 10 mg/kg + Fulvest 5 mg/wk | LEE 75mg/kg + RAD001 10 mg/kg + Fulvest 5 mg/wk |
|---|---|---|---|---|---|---|---|---|---|Everolimus + Ribo + Fulv
Treatment withdrawn
Days
Alpel + Ribo + Fulv
### Chart
| Category | Vehicle Control | LEE 75mg/kg + BYL719 35 mg/kg + Fulvest 5 mg/wk | LEE 75mg/kg + BYL719 35 mg/kg + Fulvest 5 mg/wk | LEE 75mg/kg + BYL719 35 mg/kg + Fulvest 5 mg/wk | LEE 75mg/kg + BYL719 35 mg/kg + Fulvest 5 mg/wk | LEE 75mg/kg + BYL719 35 mg/kg + Fulvest 5 mg/wk | LEE 75mg/kg + BYL719 35 mg/kg + Fulvest 5 mg/wk | LEE 75mg/kg + BYL719 35 mg/kg + Fulvest 5 mg/wk | LEE 75mg/kg + BYL719 35 mg/kg + Fulvest 5 mg/wk |
|---|---|---|---|---|---|---|---|---|---|Tumor Volume mm3
Treatment withdrawn
Vehicle Control
Vehicle Control
